# Prevalence of AZFс Y chromosome microdeletions and association with spermatogenesis in Russian men from the general population

**DOI:** 10.18699/vjgb-24-86

**Published:** 2024-11

**Authors:** L.V. Osadchuk, G.V. Vasiliev, M.K. Ivanov, M.A. Prasolova, M.A. Kleshchev, A.V. Osadchuk

**Affiliations:** Institute of Cytology and Genetics of the Siberian Branch of the Russian Academy of Sciences, Novosibirsk, Russia; Institute of Cytology and Genetics of the Siberian Branch of the Russian Academy of Sciences, Novosibirsk, Russia; Joint Stock Company Vector Best, Novosibirsk region, Russia; Institute of Cytology and Genetics of the Siberian Branch of the Russian Academy of Sciences, Novosibirsk, Russia Joint Stock Company Vector Best, Novosibirsk region, Russia; Institute of Cytology and Genetics of the Siberian Branch of the Russian Academy of Sciences, Novosibirsk, Russia; Institute of Cytology and Genetics of the Siberian Branch of the Russian Academy of Sciences, Novosibirsk, Russia

**Keywords:** AZFc deletions of the Y chromosome, spermatogenesis, male fertility, general population, AZFс-микроделеции Y-хромосомы, сперматогенез, мужская фертильность, общая популяция

## Abstract

The Y chromosome contains a set of genes with testis-specific expression that are responsible for the development of testes and spermatogenesis, and it is the most important target in the search for genetic causes of male infertility. Most of these genes are located in the “azoospermia factor” AZF locus (regions AZFa, AZFb, and AZFc) on the long arm of the Y chromosome. Microdeletions of the Y chromosome, leading to the removal of the entire AZF locus as well as one or more regions (complete deletions), are one of the leading causes of spermatogenesis impairment and infertility. However, the role of partial AZFc deletions (gr/gr, b2/b3, b1/b3) in spermatogenesis failure is unclear, and their impact on spermatogenesis varies between populations. The aim of the present study was to assess the frequency of various types of AZFc microdeletions and to search for associations with spermatogenesis parameters in men of Slavic ethnicity from the general Russian population (n = 700, average age 25.8 years). To identify AZF microdeletions, the presence/absence of 15 STS markers was analyzed using multiplex real-time polymerase chain reaction. Age, weight, height, and the volume, concentration, total count, proportion of motile and morphologically normal spermatozoa in the ejaculate were recorded for all participants. In the studied sample, 19.9 % (139/700) of men were found to have AZFc microdeletions, of which 16.7 % (117/700) were carriers of a partial b2/b3 deletion, 3.0 % (21/700) had a partial gr/gr deletion, and 0.14 % (1/700) had a complete b2/b4 deletion. Neither AZFa nor AZFb microdeletions nor other types of AZF deletions were detected. The overall frequency of all types of AZFc deletions, as well as each type of partial microdeletion, b2/ b3 and gr/gr, did not differ in the groups of azoospermia, severe oligozoospermia (≤5.0 mill/ml), oligozoospermia (5.0 < SC <16.0 mill/ml), and normal sperm concentration (≥16.0 mill/ml). Comparison of semen parameters in groups with different types of partial AZFc deletions and the control group (without deletions) also did not reveal significant differences. Thus, partial AZFc microdeletions b2/b3 and gr/gr do not have a significant impact on spermatogenesis in Slavic men. It is suggested that in Slavs, partial AZFc microdeletions b2/b3 and gr/gr are fixed in Y haplogroups N3 and R1a, respectively, and their negative impact on spermatogenesis is balanced by other genetic factors. The higher frequency of partial AZFc deletions (19.7 %) in Slavs compared to European populations (7.3 %) established in our study may be explained by the widespread distribution of these Y haplogroups in the Slavic population of Russia.

## Abstract (ru)

Y-хромосома содержит набор генов, имеющих тестис-специфическую экспрессию, ответственных за развитие яичек и сперматогенез, и является наиболее важной мишенью в поиске генетических причин мужского бесплодия. Большинство из этих генов расположены в локусе «фактора азооспермии» AZF (регионы AZFa, AZFb и AZFc) на длинном плече Y-хромосомы. Микроделеции Y-хромосомы, приводящие к удалению всего локуса AZF, а также одного или нескольких регионов (полные делеции), являются одной из ведущих причин нарушения спер- матогенеза и бесплодия, однако роль частичных AZFc-делеций (gr/gr, b2/b3, b1/b3) в нарушении сперматогенеза не ясна, а влияние на сперматогенез варьирует между популяциями. Цель настоящего исследования состояла в оценке частоты различных типов AZFс-микроделеций и поиске ассоциаций с параметрами сперматогенеза у мужчин славянской этнической группы из общей российской популяции (n = 700, средний возраст 25.8 года). Для выявления AZF-микроделеций анализировали наличие/отсутствие 15 STS-маркеров методом мультиплекс- ной полимеразной цепной реакции в режиме реального времени. У всех участников записывали возраст, вес, рост, оценивали объем, концентрацию, общее количество, долю подвижных и морфологически нормальных сперматозоидов в эякуляте. В исследуемой выборке выявлены 19.9 % (139/700) мужчин с микроделециями AZFс региона, из них 16.7 % (117/700) являлись носителями частичной делеции b2/b3, 3.0 % (21/700) – частичной де- леции gr/gr, 0.14 % (1/700) – полной делеции b2/b4. Не обнаружены AZFa и AZFb микроделеции и другие типы AZF-делеций. Суммарная частота всех типов AZFс-делеций, а также каждого типа частичных микроделеций b2/ b3 и gr/gr не различалась в группах азооспермии, тяжелой олигозооспермии (≤5.0 млн/мл), олигозооспермии (5.0 < КС < 16.0 млн/ мл) и нормальной концентрации сперматозоидов (≥16.0 млн/мл). Сравнение спермиологи- ческих показателей в группах с различными типами частичных AZFc-делеций и контролем (без делеций) тоже не выявило достоверных различий. Таким образом, частичные AZFc-микроделеции b2/b3 и gr/gr не оказывают существенного влияния на сперматогенез у славянских мужчин. Предполагается, что у славян частичные AZFс- микроделеции b2/b3 и gr/gr фиксированы в Y-гаплогруппе N3 и R1a соответственно, а их негативное влияние на сперматогенез уравновешивается другими генетическими факторами. Установленная в нашей работе более высокая частота частичных AZFc-делеций (19.7 %) у славян по сравнению с европейскими популяциями (7.3 %) также может объясняться широким распространением этих Y-гаплогрупп в славянской популяции России

## Introduction

The prevalence of male infertility in the general population is 7–12 % (Krausz et al., 2018; Cioppi et al., 2021), and in the Russian Federation 10–15 % of married couples suffer from infertility depending on the region (Lebedev et al., 2019). A number of genetic variants that negatively affect male fertility are known, and this list is continuously expanding (Cioppi et al., 2021). The Y chromosome is the most important molecular genetic target in the search for genetic causes of male infertility and subfertility (Krausz, Casamonti, 2017; Colaco, Modi, 2018). The Y chromosome carries genes necessary for the normal development of the testes and testicular functions, such as sex determination and the regulation of spermatogenesis. On the Y chromosome, the AZF locus and its three regions AZFa, AZFb and AZFc are located, and Y chromosome microdeletions leading to the removal of whole AZF regions (complete microdeletions) are the second main cause of spermatogenesis impairment and infertility after Klinefelter syndrome (Krausz, Casamonti, 2017; Krausz et al., 2024). The AZF microdeletions are usually de novo mutations, the complete microdeletion rate in the general population is 1:4,000, but in men with oligozoospermia and azoospermia it is significantly higher and can be as high as 14 % (Colaco, Modi, 2018; Cioppi et al., 2021; Deng et al., 2023). Adequate diagnostic methods have been developed for testing microdeletions in the AZF locus of the Y chromosome, and screening for complete microdeletions of AZFa and AZFb has become a mandatory part of routine diagnostic examinations for men with azoospermia and severe oligozoospermia. However, the clinical and diagnostic significance of the AZFc region remains a subject of discussion (Krausz et al., 2018). An indication for testing for Y chromosome microdeletions is a sperm concentration of less than 5 mill/mL or azoospermia, which is often observed in patients with infertility (Krausz et al., 2018).

A feature of the AZF locus of the Y chromosome is the ampliconic structure and multiple copies of genes. Ampliconic sequences are more than 99 % identical and organized into eight massive palindromes. Because palindrome sequences exhibit near-complete symmetry, they tend to form hairpin-like structures and generate homologous recombination (Kuroda et al., 2020). The most common type of AZF deletion is AZFc (70–80 %), followed by AZFa (0.5–9 %), AZFb (1–7 %), and AZFb+c (1–20 %) (Krausz, Casamonti, 2017; Cioppi et al., 2021; Krausz et al., 2024). Complete AZF deletions, which entirely remove one or more AZF regions, are associated with severe spermatogenesis failure, leading to infertility, and are never found in men with normozoospermia.

The AZFa region contains two single-copy genes USP9Y and DDX3Y and retroviral sequences HERVyq1 and HERVyq2, which are flanking AZFa. Between these directional retroviral sequences, homologous recombination could occur resulting in the deletion of AZFa, azoospermia, and Sertoli cell-only syndrome. The AZFb region contains 32 gene copies and transcription units. With a complete deletion of AZFb, the DNA segment including all 32 copies of genes and transcription units is removed, leading to maturation arrest and azoospermia. The AZFb and AZFc regions are partly overlapping, and complete AZFb or AZFb+c deletions are associated with Sertoli cell-only syndrome and azoospermia (Kuroda et al., 2020; Cioppi et al., 2021).

The AZFc region contains 12 genes in a variable number of copies for a total of 32 transcription units, which are expressed only in the testis and most often undergo deletions (Colaco, Modi, 2018; Cioppi et al., 2021; Krausz et al., 2024). A complete AZFc deletion (b2/b4) occurs as a result of homologous recombination between amplicon b2 and b4 and is characterized by spermatogenic impairment, ranging from severe oligozoospermia to azoospermia. However, in a significant number of cases, it is accompanied by residual spermatogenesis (Krausz et al., 2024). The AZFc locus contains the DAZ gene family that is a key determinant of spermatogenesis and consists of four copies (DAZ1–4). The DAZ gene contains an RNA-binding protein, which indicates the participation of genes of this family in mRNA translation and, apparently, in the differentiation of spermatogenic cells and meiotic division. Copies of the DAZ gene are distributed across two different clusters (DAZ1/2 and DAZ3/4), and their expression is observed at all stages of germ cell development (Colaco, Modi, 2018). The AZFc region is rich in amplicons, therefore, it is predisposed to a number of rearrangements, including partial deletions or duplications, as well as deletions with subsequent duplication. However, their effects on spermatogenesis are not yet clear and are actively discussed (Krausz, Casamonti, 2017). If partial deletions of AZFa and AZFb are extremely rare and are associated with reduced sperm production, then the role of partial deletions of AZFc (the most common are gr/gr, b2/b3, b1/b3) in spermatogenesis is controversial and the association with spermatogenesis varies greatly (from normozoospermia to azoospermia), but they may be compatible with natural conception or successfully overcome by assisted reproductive technologies (Bansal et al., 2016a, b).

The gr/gr partial AZFc deletion removes almost half of the AZFc gene content, including two copies of the DAZ gene (DAZ1/DAZ2 or DAZ3/DAZ4) and one copy of the BPY2 and CDY1 gene, representing a risk factor of spermatogenic impairment (Bansal et al., 2016b; Krausz et al., 2024). The phenotypic expression of the gr/gr deletion varies from azoospermia to normal sperm concentration, the cause of which is not yet clear. Since some gr/gr deletions are followed by duplications restoring the gene dosage, it is the gene copy number that may be the causal factor modulating sperm production. Geographic and ethnic differences in the frequency and clinical implications of the gr/gr deletion have been found, suggesting that Y chromosomal background can affect the testicular phenotype (Krausz, Casamonti, 2017). Certain Y haplogroups carrying a fixed gr/gr deletion may be present at high frequency in some ethnic populations and may influence the phenotypic manifestation of deletions through as yet unknown genetic factors (Sin et al., 2010; Rozen et al., 2012; Lo Giacco et al., 2014; Mokánszki et al., 2018). In the population of Northern India, the gr/gr deletion is a risk factor for impaired spermatogenesis if this deletion is not fixed in haplogroups R and H, the most common in this region (Bansal et al., 2016b).

Partial AZFc deletions of b1/b3 or b2/b3 remove more than half of the AZFc region and 12 gene copies and transcription units each. The mechanism of b1/b3 deletion formation involves a homologous recombination between sister chromatids or within a chromatid. Due to its low frequency, the effect of b1/b3 deletion on spermatogenesis remains unclear, but some authors find an increased risk of severe spermatogenic failure in men with a b1/b3 deletion (Krausz, Casamonti, 2017). The b2/b3 deletion removes over half of the AZFc, including two copies of DAZ and one copy of CDY1. The molecular mechanism of the b2/b3 deletion is complex, since it is preceded by an inversion and results in the retention of two DAZ gene copies, one BPY2 gene and one CDY1 gene. A high frequency of the b2/b3 deletion is observed in populations of Northern Eurasia, where it is fixed in Y haplogroup N and it is not a risk factor for impaired spermatogenesis and infertility. However, it may increase the risk of spermatogenic loss and infertility when occurring outside this haplogroup, for example in Mongoloid, East Asian and African populations (Rozen et al., 2012; Bansal et al., 2016a; Colaco, Modi, 2018; Hallast et al., 2021).

In clinical practice, testing for the presence of AZF deletions on the Y chromosome is recommended for infertile men with azoospermia and severe oligozoospermia for diagnostic purposes. This can, in some cases, help to identify the genetic cause of impaired spermatogenesis. The diagnosis of Y-chromosome deletions also has prognostic value and allows to resolve the issue of the possibility of surgical production of sperm (micro-TESE) for subsequent IVF/ICSI. For patients with a complete AZFc deletion and azoospermia, the prognosis for obtaining sperm is favorable. In contrast, testicular biopsy using the micro-TESE method is generally ineffective for carriers of complete microdeletions in the AZFa or AZFb regions (Krausz, Casamonti, 2017; Kuroda et al., 2020).

Fertile men carrying partial AZFc deletions or infertile men with partial AZFc deletions whose partners give birth to children through assisted reproductive technologies (micro- TESE or TESA/ICSI) can transfer these AZF deletions to the progeny (Pan et al., 2018; Deng et al., 2023). Moreover, in the paper (Pan et al., 2018), in fertile fathers who were carriers of a b2/b3 deletion or a b2/b3 duplication, the sons suffered from infertility and were carriers of a complete AZFc, AZFb+c or AZFa+b+c deletion. Thus, partial deletions of the AZFc region increase the likelihood of other microstructural rearrangements within the AZFc region, which can be a risk factor for complete AZFc deletion and infertility in male offspring.

Although the relationship between spermatogenic failure, testicular phenotype and ART outcomes in men with different types of AZFc microdeletions of the Y chromosome has been extensively studied, the population frequencies of these deletions and therefore their exact contribution to male infertility and subfertility are still insufficiently understood. Genetic testing of male populations is considered a useful approach for obtaining adequate genetic information about the prevalence of genetically determined impairment of spermatogenesis, infertility and subfertility in men of a given population. This information can be used to forecast and plan preventive, diagnostic, and clinical work aimed at preserving and improving the reproductive health of the population. Such data can serve as a basis for further genetic studies on the etiology of infertility and the determination of its causes. They can provide information about the mutation spectrum associated with spermatogenesis disorders and help to define the genetic structure of demographic risks within the population.

The aim of the present study was to analyze the spectrum and prevalence of AZF microdeletions on the Y chromosome and to search for associations with semen parameters in Slavic men from the general Russian population

## Materials and methods

Young Slavic men (Belarusians, Ukrainians, Russians) (n = 700) from five Russian cities participated in the study: Arkhangelsk (n = 77), Novosibirsk (n = 324), Kemerovo (n = 205), Ulan-Ude (n = 69), Yakutsk (n = 25). The study population also included descendants of mixed marriages between Russians and Belarusians, Ukrainians, Poles (7.1 %). The study design and standardized recruitment protocol had been described earlier in more detail (Osadchuk et al., 2021, 2022). Men from the general population, regardless of fertility status, participated in the study. All participants were either born or had lived for at least 3–5 years in the cities where the study was conducted. Most participants were students or employees of higher educational institutions at the time of the survey and had not previously consulted an andrologist. All participants were volunteers and did not receive financial compensation. The men completed questionnaires that included questions about their age, place of birth, nationality, profession, type of work, military service, smoking, alcohol consumption, and past and current diseases. Ethnic background was assessed for up to two generations – for the participant, their parents, and both maternal and paternal grandparents. All men included in the study gave informed consent to participate in the examination. The Ethics Committee of the Federal Research Center Institute of Cytology and Genetics of the Siberian Branch of the Russian Academy of Sciences approved the study (protocol No. 160 dated 17.09.2020).

During the examination, the men were examined by a urologist-andrologist, medical histories were collected, current disorders of the urogenital system were diagnosed, and the results of the examination were recorded in the examination protocol. Each volunteer was given a preliminary andrological diagnosis. The age of all participants was documented, and their height (cm) and body weight (kg) were measured. The bitesticular volume (BTV) (ml) was estimated by a Prader orchidometer. Exclusion criteria included acute diseases, taking medications or undergoing procedures affecting sperm quality (such as anabolic steroids, antibiotics and others). A preliminary condition for participation was abstinence from sexual intercourse for 2–7 days before the study. Semen samples for further laboratory analysis were collected by the participants in a specialized laboratory room through masturbation into single-use sterile plastic containers. The period of sexual abstinence was 4 days (median).

The semen samples were analyzed according to the WHO laboratory manual (WHO…, 2010, 2021). The sperm concentration was assessed using Goryaev’s hemocytometer after staining an ejaculate aliquot with trypan blue. The proportion of motile sperm with progressive straight-line movement and velocity above 25 and 2–25 μm/s (categories A and B, respectively) was assessed using the sperm analyzer SFA–500-2 (“Biola”, Russia). The analysis of sperm morphology was conducted according to WHO guidelines (WHO…, 2021). Ejaculate smears were stained using commercially available Diff-Quik kits (“Abris+”, Russia). The first 200 spermatozoa were examined for morphology with an optical microscope Axio Skop.A1 (Carl Zeiss, Germany) at ×1000 magnification with oil immersion. Sperm dimensions were measured using an ocular micrometer. Sperm morphology evaluations were done in duplicates in random and blinded order by a trained staff member. To determine the Teratozoospermia Index (TZI), the total number of identified morphological defects was divided by the number of morphologically abnormal spermatozoa.

Genomic DNA was extracted from peripheral blood leukocytes using a widely accepted phenol-chloroform method. Detection of microdeletions in the AZF locus was performed by multiplex polymerase chain reaction (PCR) with hybridization-fluorescence detection of PCR products in real-time using a CFX96 DNA amplifier (Bio-Rad, USA). In the first stage, to identify deletions, the presence or absence of 13 STS markers was analyzed using “RealBest-Genetics AZF-microdeletions” commercial kits (Vector-Best, Novosibirsk). The amplification reaction protocol included: stage 1: 50 °C – 2 min; stage 2: 95 °C – 2 min; stage 3: 50 cycles (94 °C – 10 sec, 60 °C – 20 sec). The following STS markers were investigated: sY86, sY84, sY615 – for the AZFa region; sY127, sY134, sY142 – for the AZFb region; sY1196, sY1191, sY254, sY255, sY1291, sY1206, sY1125 – for the AZFc region. Partial AZFc deletions b2/b3 and gr/gr were indicated by the absence of markers sY1191 and sY1291, respectively; complete AZFc deletion b2/b4, by the absence of markers sY1191, sY1206, sY1291, sY254, and sY255; partial AZFc deletion b1/b3, by the absence of markers sY1191, sY1196, and sY1291. STS marker typing was conducted using five reaction mixtures (RM). RM1 included markers for the SRY gene (sex-determining gene), sY134, sY84, sY254; RM2, for the HMDS gene (a gene for additional DNA control), sY127, sY86, sY255; RM3, for the HMDS gene, sY142, sY615; RM4, for sY1191, sY1196, sY1206, sY1125; RM5, for the SRY gene, sY1296. Genotyping of the SRY gene and the autosomal HMBS gene (quality control of the material collection) was conducted as an internal control.

In the second stage, for the most common partial microdeletions of the AZFc region – b2/b3 (marker sY1191) and gr/ gr (marker sY1291) – verification was performed using two additional STS markers, sY1192, sY1189, which are closely linked to the corresponding markers. Detection of STS markers sY1192 and sY1189 was carried out by their amplification. The amplification reaction protocol was two-step: 50 °C for 2 min, 95 °C for 2 min, 40 cycles (95 °C for 10 sec, 66 °C for 20 sec) followed by electrophoresis in a 1.2 % agarose gel, staining with ethidium bromide, and visualization under ultraviolet light.

A statistical analysis of the obtained data was performed using the statistical package STATISTICA (version 8.0). For all studied parameters, the mean (SD) was calculated. The Kolmogorov–Smirnov test was used to confirm the normal distribution of quantitative variables. Since most parameters did not follow a normal distribution, differences in the studied anthropometric and spermiological parameters between groups with different sperm concentrations or between groups with different types of partial AZFc microdeletions were determined using the Kruskal–Wallis one-way analysis of variance (Kruskal–Wallis ANOVA) and analysis of covariance (ANCOVA). In the latter case, spermiological parameters were adjusted for age and abstinence period. For pairwise comparison of groups, Duncan’s test was applied. Comparisons of the frequencies of AZFc microdeletions between groups were conducted using the chi-square (χ2) test. A p-value < 0.05 was considered statistically significant.

Characteristics of the Slavic study population. According to the results of a physical examination and medical history, the study population consist of 16 (2.3 %) individuals with testicular hypoplasia, 50 (7.1 %) with grade II and III varicocele, 8 (1.1 %) who underwent surgery for cryptorchidism, and 43 (6.4 %) who underwent varicocelectomy. Among 700 participants, 2.7 % suffered from azoospermia, 4.7 %, from severe oligozoospermia, 10.6 %, from moderate oligozoospermia, but 82.0 % had normal sperm concentration (SC) according to WHO recommendations (WHO…, 2021).

Based on sperm concentration (SC), participants were stratified into four groups: 1) SC = 0 mill/mL (azoospermia, absence of spermatozoa in the ejaculate); 2) SC ≤ 5.0 mill/mL (severe oligozoospermia); 3) 16.0 > SC > 5.0 mill/mL (moderate oligozoospermia); 4) SC ≥ 16.0 mill/mL (normal sperm concentration). Anthropometric and spermiological indicators of men in groups with varying sperm concentrations are presented in Table 1. No differences were found between the groups in terms of age, anthropometric parameters, and ejaculate volume. The total sperm count, sperm concentration, percentage of motile and morphologically normal sperm, and BTV in the group with normal sperm concentration were significantly ( p < 0.05) higher, whereas the TZI was significantly lower ( p < 0.05) compared to both oligozoospermia groups, which did not differ from each other in these parameters.

**Table 1.**
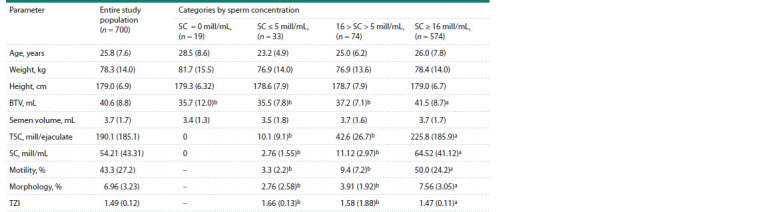
Anthropometric and spermiological parameters of men in the entire study population and after stratification into categories by sperm concentration Note. Data are presented as mean (SD). BTV – bitesticular volume (paired testicular volume); TSC – total sperm count per ejaculate; SC – sperm concentration; motility – percentage of motile spermatozoa in categories A+B; morphology – percentage of morphologically normal spermatozoa; TZI – teratozoospermia index. а, b Comparisons with different superscripts within variables were significant (p <0.05).

## Results

Prevalence of different types of AZFc microdeletions in the Slavic study population

Since the study population included Slavs residing in 5 cities of Russia, a comparison of the prevalence of AZFc microdeletions in each city group was conducted (Table 2). Statistical analysis did not reveal significant regional differences in the frequency of partial deletions b2/b3 and gr/gr (χ2 8 = 6.46; p < 0.595).

**Table 2.**
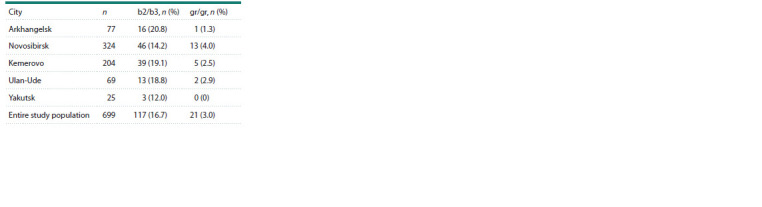
Frequency of partial AZFc microdeletions (b2/b3 and gr/gr) in the Slavic groups from the studied cities of Russia

From the study population, two groups were formed: one with normal sperm parameters (normozoospermia, n = 417) and the other with impaired sperm parameters (pathozoospermia, n = 282) in accordance with reference values of the WHO (WHO…, 2021). The latter had either a sperm concentration of less than 16 mill/mL, a proportion of motile spermatozoa (categories A+B) less than 30 %, a proportion of morphologically normal spermatozoa less than 4 %, or any combination of these deviations. The groups were compared for the frequency of AZFc microdeletions b2/b3 and gr/gr, with the results presented in Table 3. Statistical analysis did not reveal significant differences in the frequency of b2/b3 and gr/gr deletions between the normozoospermia and pathozoospermia groups (χ2 2 = 0.21; p < 0.90). Consequently, pathozoospermia is not associated with an increased frequency of either type of partial AZFc deletions.

**Table 3.**
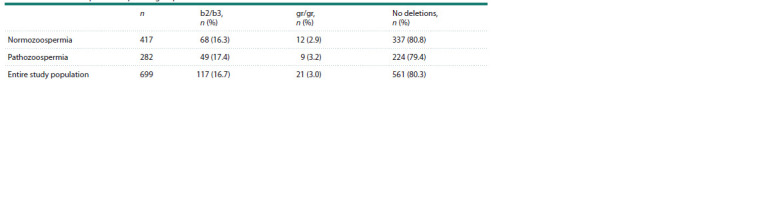
Frequency of partial AZFc microdeletions (b2/b3 and gr/gr) in the entire Slavic study population and in the normo- and pathozoospermia groups Note. Normozoospermia – sperm concentration ≥ 16.0 mill/mL, proportion of motile sperm (categories A+B) ≥ 30 %, proportion of morphologically normal sperm ≥ 4.0 % (WHO…, 2021); pathozoospermia – concentration, proportion of progressively motile and morphologically normal sperm below reference values (either each indicator or any combination thereof). The carrier of the complete AZFc microdeletion b2/b4 was not included in the table.

The prevalence of various types of AZFc deletions in the entire study population, as well as in groups with different sperm concentrations, is presented in Table 4. Y chromosome microdeletions were detected in 139 (19.9 %) out of 700 men; no AZFa, AZFb, and AZFb+c deletions were found among them. A complete AZFc deletion (b2/b4) was found in one man (0.1 %) and it was associated with azoospermia. The following types of partial AZFc deletions were identified: gr/gr in 21 (3.0 %) and b2/b3 in 117 (16.7 %) men. The combined frequency of both types of AZFc deletions did not differ between groups with different sperm concentrations (χ2 3 = 1.10, p = 0.78), neither did the frequencies of specific types of partial AZFc deletions – gr/gr (χ2 3 = 4.73, p = 0.19) and b2/b3 (χ2 3 = 2.14, p = 0.54). Therefore, no differences were found in the frequency of AZFc deletions gr/gr and b2/ b3 between groups with different sperm concentrations, indicating the absence of an impact of these deletions on sperm production in Slavic men.

**Table 4.**
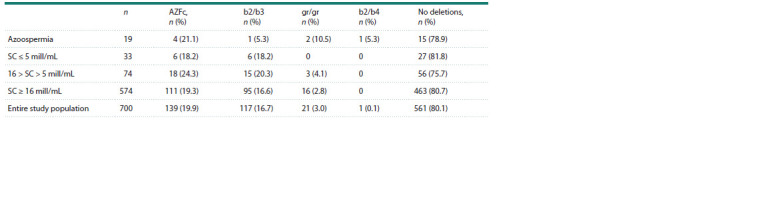
Frequency of various types of AZFc deletions in the entire Slavic study population and in the groups stratified by sperm concentration Note. Azoospermia – no spermatozoa in the ejaculate; SC – sperm concentration.

Analysis of associations of partial AZFc microdeletions and spermiological parameters

A comparison of spermiological indicators between men with partial AZFc microdeletions (b2/b3 and gr/gr) and those without microdeletions was made. The results are shown in Table 5. No significant differences were found in any spermiological parameters between the carriers of b2/b3 and gr/gr deletions and men without deletions. Therefore, our study did not establish any impact of partial AZFc deletions (b2/b3 and gr/gr) on the examined semen parameters in Slavic men.

**Table 5.**
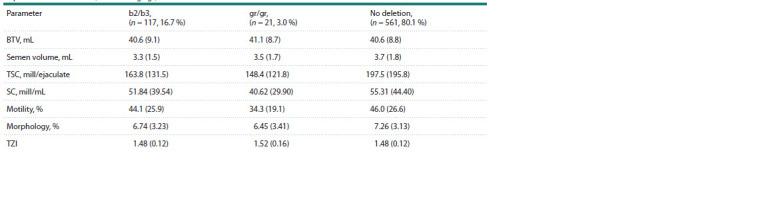
Spermiological parameters in Slavic men with different types of partial AZFc deletions (b2/b3 and gr/gr) Note. Data are presented as mean (SD); BTV – bitesticular volume; TSC – total sperm count per ejaculate; SC – sperm concentration; motility – proportion of motile sperm of category A+B; morphology – proportion of morphologically normal sperm; TZI – teratozoospermia index. Motility, morphology, and TZI parameters are calculated excluding participants with azoospermia and severe oligozoospermia

## Discussion

The global prevalence of complete AZF deletions, i. e., those that fully remove one or more regions, among infertile men is 7.5 %, which is significantly higher than in the general population – 0.025 % (Colaco, Modi, 2018; Cioppi et al., 2021). In a multi-ethnic group of Russian infertile men with azoospermia/ oligozoospermia, the prevalence of complete AZF deletions ranged from 7.5 to 12 % (Chernykh et al., 2006; Mikhaylenko et al., 2019), which is close to the rates observed in other European and Asian countries. In our Slavic study population from the general Russian population, only one man was identified with a complete AZFc b2/b4 deletion and azoospermia, confirming the low frequency of this type of AZF microdeletions in the general population. A significant increase in sample size is required to determine the prevalence of complete AZFc microdeletion among Slavs.

Among Slavic men from European countries, the prevalence of complete deletions of various AZF regions is lower than that in Russian men. For example, the frequency of complete AZF microdeletions in Slovakia among men with azoospermia was 3.35 % (Behulova et al., 2011); in Slovenia among subfertile men, 4.4 % (Peterlin et al., 2002); and in Macedonia among infertile men, 4.1 % (Plaseski et al., 2006). In the non- Slavic population of Europe, the frequency of complete AZF microdeletions in infertile men varied within the same range of 2.4–4.0 % (Lo Giacco et al., 2014; Mokánszki et al., 2018; Johnson et al., 2019).

In Asian countries, higher frequencies of complete AZF microdeletions have been identified in infertile patients with azoospermia/oligozoospermia compared to European countries: in China, 10.7–12.9 % (Liu et al., 2019; Fu et al., 2023); in Japan, 7.5 % (Iijima et al., 2020); in Turkey, 9.6–25.0 % (Akbarzadeh Khiavi et al., 2020); in Iran, 12.1–20.6 % (Bahmanimehr et al., 2018); in India, 10.0–16.1 % (Waseem et al., 2020). Despite extensive study on the geographic and ethnic variability in the frequency of complete Y-chromosome microdeletions, the underlying causes of this variability remain unknown but are largely thought to be influenced by the inappropriate selection criteria of patients

Complete deletions of the AZF regions of the Y chromosome are rare, with most (over 80 %) being partial microdeletions of the AZFc region (Krausz, Casamonti, 2017; Cioppi et al., 2021). In our Slavic study population from the general Russian population, two types of partial AZFc deletions were identified – the gr/gr and b2/b3 deletions. The combined prevalence of these types of deletions was 19.7 %, with the frequency of the gr/gr deletion being 3.0 %, and that of the b2/b3 deletion being 16.7 %. Notably, the frequency of the b2/b3 and gr/gr deletions in the normozoospermic group did not differ from that in the pathozoospermic group. Since these types of deletions (b2/b3 and gr/gr) are found in men with normozoospermia, they are not markers of impaired spermatogenesis. It should be noted that information on the frequency of these types of partial AZFc deletions in men from the general population is sparse, but there are data on the frequency of these deletions in Russian infertile men with azoo-/oligozoospermia and in Russian fertile men with normozoospermia (Table 6). In a multi-ethnic Russian group of fertile men with normozoospermia (Barkov et al., 2014) or in men from infertile married couples with normozoospermia (Zobkova et al., 2017), the frequencies of the b2/b3 and gr/gr deletions are close to our data (Table 6). In both studies, no differences in the frequency of the b2/b3 deletion were found between the normozoospermic and azoo-/oligozoospermic groups, which also aligns with our conclusions. In Russian fertile men (sperm data not specified), the frequencies of the b2/b3 and gr/gr deletions practically coincide with our data (Chernykh et al., 2022). In Russian studies (Barkov et al., 2014; Zobkova et al., 2017), the higher frequency of the gr/gr deletion in men with infertility or from infertile couples with pathozoospermia compared to those with normozoospermia (although differences are not statistically significant) draws attention to itself, which may be due to the multi-ethnic composition of the groups studied and, accordingly, different genetic background of the Y chromosome. Collectively, the data confirm that the most common types of partial AZFc deletions in Russian men are b2/b3 and gr/gr, and the practical lack of differences in the frequency of these deletions between men with normozoospermia and pathozoospermia indicates the absence of negative effects of partial b2/b3 and gr/gr deletions on spermatogenesis

**Table 6.**
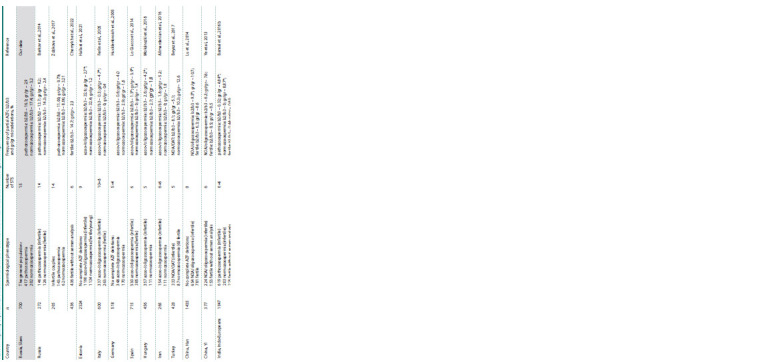
Frequency of partial AZFc microdeletions in the Y chromosome in men of different ethnic origin and region of residence Note. NOA – non-obstructive azoospermia; OAT – oligoastenotheratozoospermia. * Significant differences between groups with pathozoospermia and normozoospermia (fertile) (p <0.05).

Interestingly, in Estonia, fertile men with normozoospermia or with infertility and pathozoospermia have a higher frequency of b2/b3 deletions compared to our data and a similar frequency of gr/gr deletions (Hallast et al., 2021) (Table 6). About two-thirds of Estonian men carrying the gr/gr deletion belonged to haplogroup R1, and almost all (99.4 %) of the men carrying the b2/b3 deletion belonged to Y-haplogroup N3. The frequency of the b2/b3 deletion did not differ between the pathozoospermic and normozoospermic groups, which is consistent with the conclusions of our study. However, the frequency of the gr/gr deletion was significantly higher in the pathozoospermic group compared to the normozoospermic group. At the same time, andrological parameters in men with either b2/b3 or gr/gr deletions and without deletions did not differ.

In men from other European countries, a lower prevalence of partial AZFc deletions is observed, with most AZFc deletions represented by the gr/gr deletion (Table 6). In Italy (Ferlin et al., 2005), Germany (Hucklenbroich et al., 2005), Spain (Lo Giacco et al., 2014), and Hungary (Mokánszki et al., 2018), the frequency of the b2/b3 deletion among patients with normozoospermia ranged from 0 to 2.7 %, and among those with azoospermia/oligozoospermia, from 0.3 to 2.6 %, while the frequency of the gr/gr deletion among men with normozoospermia ranged from 0.4 to 1.8 % and among those with azoo-/oligozoospermia, from 3.9 to 4.7 %. The results of these European studies suggest that the gr/gr deletion is a genetic cause of reduced sperm production, although some authors believe that the gr/gr deletion is only a risk factor predisposing to impaired spermatogenesis.

In Asian countries, there is greater geographical heterogeneity in the frequency of partial AZFc deletions compared to Russia (Table 6). For example, in Iran, the frequency of the b2/b3 deletion among men ranged from 0 to 1.8 %, which is significantly lower than the data from Russia, while the gr/gr deletion ranged from 1.8 to 5.2 %, which is close to the Russian data, and no negative impact of these types of partial deletions on spermatogenesis was identified (Alimardanian et al., 2016). In Turkish men, the frequency of partial b2/b3 deletions is significantly lower compared to Russian men, but both types of deletions (b2/b3 and gr/gr) are not associated with infertility and reduced sperm production (Beyaz et al., 2017). Conversely, screening for partial gr/gr and b2/b3 deletions in the Han Chinese population showed that the frequency of the b2/b3 deletion was significantly higher in patients with infertility and azoo-/oligozoospermia compared to fertile men with normozoospermia, indicating an association of this deletion with impaired spermatogenesis (Lu et al., 2014). However, in Chinese men from another ethnic group, Yi, no such association was found (Ye et al., 2013), underscoring the importance of considering the ethnic composition of the population when studying the effects of partial AZFc deletions in the Y chromosome on spermatogenesis. In the Indian population, the frequency of the b2/b3 deletion was 40–45 times lower, and the frequency of the gr/gr deletion was 2–3 times higher compared to the Russian populations, with gr/gr deletions being the most common and significant among partial AZFc deletions, reducing sperm concentration and increasing the risk of infertility (Bansal et al., 2016b) (Table 6).

The provided data support the previously expressed idea (Rozen et al., 2012) that the geographical and ethnic origin of a population may influence the frequency of partial AZFc deletions b2/b3 and gr/gr. In that study, the prevalence of partial gr/gr and b2/b3 microdeletions was evaluated in 20,884 men from five populations (India, Poland, Tunisia, USA, Vietnam). It was found that the frequency of the gr/gr partial deletion varied from 2.1 % (USA) to 15 % (Vietnam), and b2/b3, from 0.5 % (India) to 2.2 % (Poland). The authors suggested that ethnogeographic differences in the frequency of the b2/b3 deletion are likely due to differences in the prevalence of Y haplogroup N1, the high prevalence of the gr/gr deletion might be due to the prevalence of haplogroup D2a chromosomes (containing these deletions), which corresponds to the hypothesis of the relationship between the frequency of partial AZFc deletions and the genetic background of the Y chromosome.

Analysis of spermiological phenotypes in carriers of Y chromosome microdeletions shows that while complete deletions of one or more AZF regions are associated with impaired spermatogenesis and are specific genetic markers of spermatogenesis failure and infertility, partial AZFc deletions exhibit heterogeneity in terms of spermiological phenotype and are often only risk factors predisposing to pathozoospermia and infertility. In our study population of Slavic men from the general population, no negative effects of b2/b3 and gr/ gr deletions on spermatogenesis were detected. In another Russian study, among men with infertility who were carriers of gr/gr and b2/b3 microdeletions (3.5 and 7.9 % of the total number examined), sperm concentration was 12.2 ± 7.1 and 30.3 ± 5.3 mill/mL, respectively, i. e., in carriers of the gr/gr deletion, it was below the reference value of the norm (Chernykh et al., 2014). The negative association of the gr/gr deletion with sperm concentration may be due to the multiethnic composition of the group and, consequently, different genetic backgrounds of the Y chromosome. The gr/gr microdeletion did not show a statistically significant association with spermatogenesis failure in other Slavic populations, such as Bulgarians (Levkova et al., 2020) and Macedonians (Kuzmanovska et al., 2019).

In some Mongoloid populations, no negative effects of the gr/gr deletion on spermatogenesis have been established, for example, in Han Chinese (Yang et al., 2010) or Japanese (Sin et al., 2010), if the gr/gr deletion is fixed in the prevalent haplogroups Q1 and D2b, respectively, which plays a role in the clinical manifestation of the deletion. However, in Koreans, the gr/gr deletion causes spermatogenesis impairment if it is not fixed in the prevalent haplogroup YAP (the precursor of haplogroup D2b), and a normal testicular phenotype is observed when it is in haplogroup YAP (Choi et al., 2012). In the African population of Tunisia, partial gr/gr and b2/b3 deletions are not associated with spermatogenesis failure, which is due to the fixation of these deletions in haplogroup E3b2, which is widespread in North Africa (Ghorbel et al., 2012). Recall that in European populations, the gr/gr deletion is associated with spermatogenesis impairment, particularly in Spaniards (Lo Giacco et al., 2014), Italians (Ferlin et al., 2005), and Hungarians (Mokánszki et al., 2018).

Many authors conclude that the influence of the partial AZFc b2/b3 deletion on spermatogenesis and fertility largely depends on the ethnic composition of the studied population, and the frequency and phenotypic effect are determined by the origin of the Y chromosome. The b2/b3 deletion is a risk factor for spermatogenesis impairment in East Asian and African populations but not in European or South Asian populations (Bansal et al., 2016a; Colaco, Modi, 2018). In the Chinese population, the b2/b3 deletion increases the risk of spermatogenesis impairment and predisposes to the forma- tion of a complete AZFc region deletion (Lu et al., 2014). However, in the ethnic Han Chinese population from eastern China, the b2/ b3 deletion is not associated with spermatogenesis impairment, which the authors attribute to interpopulation differences in the frequencies of Y haplogroups in China (Zhang et al., 2007).

In Finno-Ugric, Balto-Slavic, and some Turkish-speaking peoples of Northern Eurasia, the partial AZFc b2/b3 deletion is fixed in Y haplogroup N, which has a high frequency (up to 90 % in some populations) (Repping et al., 2004). It has been established that the b2/b3 deletion does not cause spermatogenesis impairment in Germans (Hucklenbroich et al., 2005); Tunisians (Ghorbel et al., 2012); Iranians (Alimardanian et al., 2016); Hungarians (Mokánszki et al., 2018); and Estonians (Hallast et al., 2021). The population of ethnic Russians can also be included in this group due to the lack of association of this deletion with spermatogenesis failure, the high frequency of the b2/b3 deletion, and the wide prevalence of Y haplogroup N3 carrying this deletion, which varies between 10–19 % (Stepanov et al., 2006; Balanovska, Balanovsky, 2007; Derenko et al., 2007). It is hypothesized that the effect

of the b2/b3 deletion in haplogroup N is balanced by other genetic factors, possibly related to the Y chromosome (Repping et al., 2004).

Thus, the effects of partial AZFc deletions on spermatogenesis can depend on the lineage of the Y chromosome (the Y haplogroup carrying the deletion), increasing or decreasing the risk of spermatogenesis impairment in certain populations. Since partial deletions of the AZFc region can be fixed in specific Y haplogroups, the population frequency of partial AZFc deletions depends on the frequency of these Y haplogroups, and the impact of partial AZFc deletions on spermatogenesis may differ in the Y chromosomes of different haplogroups. For example, in Japanese men, the two most common Y haplogroups are D (34.7 %) and O (51.8 %). The gr/gr deletions were found in 33.7 % of Japanese men, but the frequency of the gr/gr deletion varied significantly depending on the Y haplogroup: it was widespread in haplogroup D (86.2 %) and much less so in haplogroup O (5.1 %), with it being phenotypically neutral in haplogroup D, meaning it did not affect spermatogenesis, while in haplogroup O, it reduced sperm concentration (Sin et al., 2010). In men from Northern Italy, a comparison of the distribution of seven Y haplogroups between a group of fertile men and patients with microdeletions did not reveal any differences, but the frequency of Y haplogroup E with the b2/b4 deletion was significantly higher compared to the control. The results suggest that some haplogroups may be more prone to AZFc b2/b4 microdeletions than others (Arredi et al., 2007).

In the population of ethnic Russians in Russia, the dominant Y haplogroup is R1a, which is the most common (over 40 %), followed by N3 (10–19 %), and I1b (13 %) (Stepanov et al., 2006; Balanovska, Balanovsky, 2007; Ilumäe et al., 2016). Although the association between the phenotypic expression of partial AZFc deletions in the Y chromosome and Y haplogroups remains a subject of discussion, considering the aforementioned facts, it appears promising to study the association of the main haplogroups R1a, N3, and I1b with spermatogenesis indicators in Slavic men. This will help to elucidate a modulatory effect of the haplogroup on the phenotypic expression of partial AZFc deletion. Special attention should be given to haplogroups N3 and R1a, which contain the b2/b3 deletion and gr/gr deletion, respectively (Repping et al., 2004; Rozen et al., 2012). The prevalence of the b2/b3 deletion in our study (16.7 %) coincides with the prevalence of haplogroup N3 in the Russian population (10–19 %); in this haplogroup, this deletion does not affect spermatogenesis, although in another haplogroup it may have a negative effect on the spermatogenic phenotype. It is assumed that in haplogroup N3, the negative impact of the deletion on spermatogenesis is balanced by other genetic factors, possibly also associated with the Y chromosome (Repping et al., 2004).

## Conclusion

In a study population of Slavic men recruited from five cities in Russia (n = 700), the spectrum and frequency of AZFc microdeletions in the Y chromosome were determined. It was found that 19.9 % were carriers of AZFc deletions, of which 16.7 % were carriers of a partial b2/b3 deletion, 3.0 % had a partial gr/gr deletion, and 0.14 % had a complete b2/b4 deletion. No AZFa and AZFb microdeletions or other types of AZF deletions were found.

The overall frequency of all types of AZFc deletions, as well as partial b2/b3 and gr/gr deletions, did not differ between the groups with normozoospermia and pathozoospermia, nor between the groups with azoospermia, severe oligozoospermia, oligozoospermia, and normal sperm concentration. Semen parameters did not differ between the groups with different types of partial AZFc deletions and the group without deletions. The data obtained indicate the absence of a pathogenic role of partial AZFc deletions in spermatogenesis of Slavic men.

## Conflict of interest

The authors declare no conflict of interest.
